# Molecular Characterization and Genetic Relatedness of Potential Enteric Pathogens Isolated From Household Drinking Water and Diarrheal Stools of HIV‐Infected Individuals: Implications for Environmental Exposure

**DOI:** 10.1002/wer.70665

**Published:** 2026-11-13

**Authors:** C. M. N. Khabo‐Mmekoa, K. Jitendra, M. N. B. Momba

**Affiliations:** ^1^ Department of Biomedical Technology, Arcadia Campus Tshwane University of Technology Pretoria South Africa; ^2^ Department of Environmental, Water and Earth Sciences, Arcadia Campus Tshwane University of Technology Pretoria South Africa

**Keywords:** 16S rRNA gene sequencing, container‐stored drinking water, diarrheal stool, enteric bacteria, HIV‐infected individuals, PCR‐RFLP fingerprinting, potential environmental exposure

## Abstract

The quality of drinking water is critical for immunocompromised populations. This study investigated the prevalence and genetic relatedness of potential enteric bacteria in household container‐stored drinking water and diarrheal stools from 164 participants (120 HIV‐infected, 44 HIV‐negative) in Ugu District, South Africa. In stool specimens, pathogen prevalence was markedly higher among HIV‐infected individuals for *Escherichia coli* (88%), *Salmonella* spp. (33%), *Shigella* spp. (27%), and *Vibrio* spp. (12%) compared to HIV‐negative controls ( *E. coli* 34%, *Salmonella* spp. 2%). Container‐stored drinking water in Boboyi showed pathogen detection frequencies exceeding 80%. Molecular analysis of 143 representative isolates (80 water, 63 stool) revealed close genetic clustering between water‐derived and clinical *E. coli* . While these findings highlight significant environmental exposure, conserved 16S rRNA sequences identify container‐stored water as a potential exposure source rather than a proven direct transmission vector, underscoring the need for improved household water safety.

## Practitioner Points

- **High Pathogen Prevalence in Container Water**: Potential enteric bacteria ( *Escherichia coli* , *Salmonella* spp., *Shigella* spp., and *Vibrio* spp.) were widely detected in household container‐stored drinking water (*n* = 638 growth‐positive samples) across rural Ugu District communities, exceeding 80% detection frequency in Boboyi.
- **Elevated Clinical Burden in Immunocompromised Individuals**: Stool specimens from HIV‐infected individuals with diarrhea (*n* = 120) exhibited significantly higher pathogen prevalence ( *E. coli* 88%, *Salmonella* spp. 33%, *Shigella* spp. 27%, and *Vibrio* spp. 12%) compared to HIV‐negative controls (*n* = 44; *E. coli* 34%, *Salmonella* spp. 2%).
- **Genetic Relatedness Demonstrates Exposure Risk**: Molecular characterization (16S rRNA PCR‐RFLP and Sanger sequencing of *n* = 143 representative isolates) revealed tight sequence clustering between water‐derived and clinical *E. coli* isolates, identifying container‐stored water as a critical potential exposure source.
- **Necessity for High‐Resolution Surveillance**: While 16S rRNA sequence similarity confirms species identification and environmental risk, high‐resolution strain typing (whole‐genome sequencing or MLST) is required to establish direct outbreak transmission pathways.
- **Actionable WASH Interventions**: To protect vulnerable immunocompromised populations, municipal water authorities and health practitioners must implement point‐of‐use (POU) household water treatment technologies, promote hygienic closed storage containers, and conduct regular microbial monitoring.

## Graphical abstract

Container‐stored drinking water in rural South Africa represents a critical potential environmental exposure pathway for enteric bacteria ( *Escherichia coli* , *Salmonella*, *Shigella*, and *Vibrio* spp.) among immunocompromised HIV‐infected individuals, underscoring the urgent need for targeted household point‐of‐use water treatment.

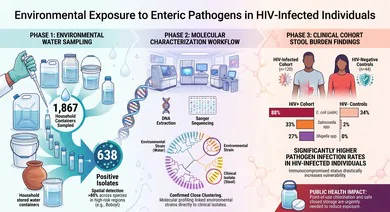

## 1 Introduction

Diarrheal disease remains a major public health concern globally, contributing significantly to morbidity and mortality, particularly among children under 5 years of age UNICEF (United Nations International Children's Emergency Fund) 2012. Estimates from the Global Burden of Disease study indicate a disproportionate mortality burden in sub‐Saharan Africa, where infrastructure and water sanitation facilities are often inadequate (Wang 2016; WHO (World Health Organization) 2019). Furthermore, global monitoring highlights that billions of people in low‐ and middle‐income regions still lack access to safely managed drinking water and basic household sanitation services (WHO 2017, 2019). Individuals living with HIV (PLWH) experience heightened susceptibility to waterborne opportunistic pathogens, enduring recurrent diarrheal episodes that contribute to chronic malnutrition, reduced quality of life, and increased mortality (Wilcox et al. 1996). Transmission of these enteric pathogens occurs primarily through the fecal–oral route via ingestion of contaminated water or food, direct contact with fecal matter, or person‐to‐person contact (Mead et al. 1999; Obi et al. 2004; Momba et al. 2006).

In South Africa, despite post‐apartheid efforts to expand municipal water infrastructure, many nonmetropolitan and rural households continue to rely on unimproved surface water (rivers and dams) or on contaminated groundwater and inadequately treated water for domestic use (Momba et al. 2006; Momba et al. 2009; Moropeng et al. 2018; National State of Water Report (NSW) 2021; Sekgobela et al. 2023). Furthermore, even when water is collected from improved sources, household container storage frequently leads to secondary microbial recontamination (Donovan et al. 2008; Abhirosh et al. 2010; WHO (World Health Organization) 2000; Sobsey 2002; Cabral 2010; UNICEF (United Nations International Children's Emergency Fund) 2011; Momba et al. 2008; Murei et al. 2023). This places rural communities at elevated risk of exposure to enteric bacterial pathogens, with severe health consequences for immunocompromised individuals prone to persistent gastroenteritis (Obi et al. 2007; UNICEF (United Nations International Children's Emergency Fund) 2011; Fauci 1999; Lubeck et al. 1993; Chaisson et al. 1998; Hayes et al. 2003; Fleury et al. 2008).

Conventional culture‐based techniques remain fundamental for bacterial isolation but are labor‐intensive and limited in distinguishing closely related taxa with overlapping phenotypic characteristics (Theron and Cloete 2002; Adzitey and Huda 2010; Yashiro et al. 2011; Stefani et al. 2015). Genotypic approaches targeting conserved genetic markers such as the 16S rRNA gene combined with restriction fragment length polymorphism (RFLP) and polymerase chain reaction (PCR) provide superior sensitivity, specificity, and discriminatory resolution (Donelli et al. 2013; Islam et al. 1998; Lampel et al. 2000; Kulkarni et al. 2002). These molecular tools facilitate high‐resolution comparative evaluations of bacterial diversity and genetic relatedness between environmental and clinical isolates (Tsen and Jian 1998; Waage et al. 1999; Rood et al. 2011; Salama et al. 2012; Njage and Buys 2015; Nandi et al. 2000; Goel et al. 2007).

While baseline investigations in Limpopo and Eastern Cape provinces have documented the occurrence of enteric bacteria in rural water and stool samples, limited information exists regarding the sequence‐level genetic relatedness of environmental and clinical bacterial isolates in KwaZulu‐Natal (Murei and Momba 2025). Therefore, this study investigated the molecular characterization and genetic relatedness of selected enteric bacteria (*Salmonella*, *Shigella*, *Vibrio*, and *Escherichia coli* ) isolated from household container‐stored drinking water and diarrheal stool specimens of HIV‐infected individuals attending Port Shepstone and Murchison hospitals in Ugu District Municipality (Obi et al. 2007; Momba et al. 2008; Thompson et al. 2004; Pester et al. 2012; van Megen et al. 2009; Steinberg and Regan 2008).

## 2 Materials and Methods

### 2.1 Description of the Study Site and Population

The study was conducted in Ugu District Municipality, located in KwaZulu‐Natal Province, South Africa, bordering the Eastern Cape Province (Figure 1). The municipality covers approximately 5866 km^2^ and serves a population of approximately 700,000. Figure 1 indicates a map displaying the geographic locations of rural study communities (Boboyi, Bomela, and Gamalakhe) where household container‐stored drinking water samples and clinical diarrheal stool specimens were collected.

**FIGURE 1:**
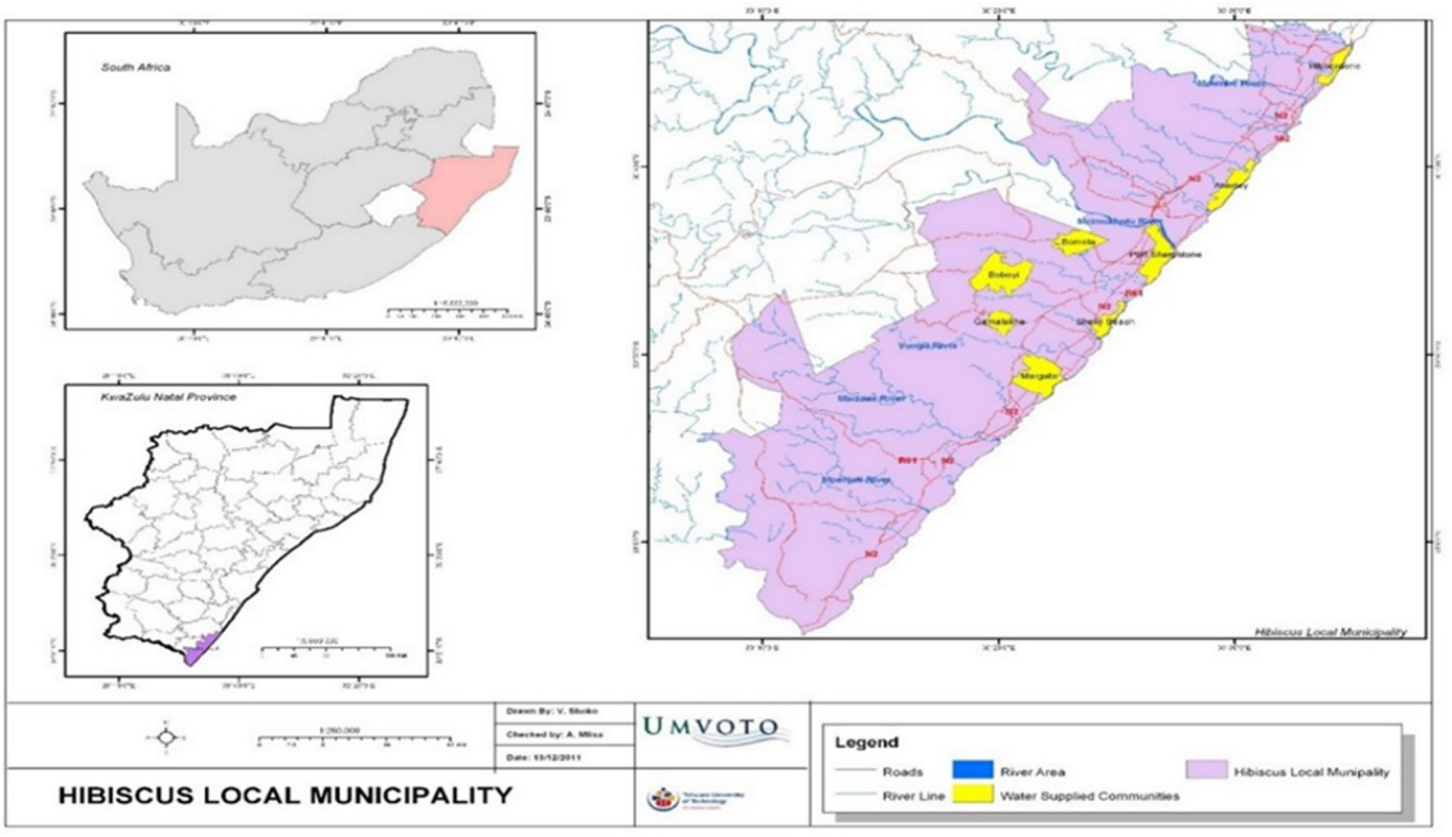
Map of the study area in Ugu District Municipality, KwaZulu‐Natal Province, South Africa. [Map displaying the geographic locations of rural study communities (Boboyi, Bomela, and Gamalakhe) where household container‐stored drinking water samples and clinical diarrheal stool specimens were collected.]

### 2.2 Scientific Ethics and Informed Consent

Ethical approval and research clearance for the study were granted by the Faculty Committee for Research Ethics (FCRE) at Tshwane University of Technology (TUT) under the reference number (2006/07/028[a]), the KwaZulu‐Natal Provincial Department of Health Research Committee under reference number (050/06), and the healthcare facility management teams at Port Shepstone and Murchison hospitals. Written informed consent was obtained from all adult participants prior to enrolment. Where applicable, consent for stool specimen collection was obtained from parents or legal guardians and facilitated by trained healthcare personnel involved in voluntary counseling and testing (VCT). For household drinking water sampling, informed consent was obtained from household owners before sample collection. All participants were informed of the purpose, objectives, and procedures of the study prior to participation.

### 2.3 Collection of Water Samples, Stool Specimens, and Sample Processing Flow

A total of 1867 drinking water samples were collected from rural and urban communities in Ugu District Municipality between January 2008 and November 2009. An additional sampling round was conducted in April 2015 to evaluate possible changes in access to drinking water and water quality over time. For households supplied through tap water systems, tap outlets were disinfected using 70% ethanol and flushed for approximately 1 min before sample collection. Drinking water samples were collected aseptically into sterile 1 L sampling bottles containing 120 mg sodium thiosulphate (Na_2_S_2_O_3_) to neutralize residual disinfectants (APHA 2001). Following collection, samples were transported on ice in insulated cooler boxes to the Boboyi Water Treatment Plant Laboratory, where initial microbiological analyses were performed within 4–6 h of collection.

A total of 164 diarrheal stool specimens were collected by hospital clinicians from patients attending Port Shepstone and Murchison hospitals. Of these, 120 specimens were obtained from individuals with confirmed HIV‐positive status and 44 from HIV‐negative individuals. HIV status was established independently through routine clinical procedures prior to enrolment in the study. To maintain participant confidentiality and anonymity, specific clinical variables (CD4 cell counts, viral load, and ART regimen) were not recorded.

Drinking water samples and stool specimens were transported separately in insulated cooler boxes to the Boboyi Water Treatment Plant Laboratory and the National Health Laboratory Service (NHLS) at Port Shepstone Hospital, respectively. Isolation of enteric bacteria was performed within 4–6 h of collection. Recovered isolates were preserved and transported under aseptic conditions to the Water Research Group Laboratory at Tshwane University of Technology for molecular analysis. Of the 1867 drinking water samples collected, 638 samples demonstrated significant bacterial growth on selective culture media and were processed further. All 164 stool specimens yielded bacterial growth and were included in downstream analysis.

To ensure complete transparency regarding isolate selection and representativeness, Table 1 outlines the sample‐processing pathway from initial collection through PCR, RFLP screening, and Sanger sequencing.

**TABLE 1:** Transparent sample‐processing pathway and isolate selection breakdown.

Processing stage	Household drinking water	Diarrheal stool specimens	Combined total
Initial collected samples/specimens	1867 samples	164 specimens (120 HIV+, 44 HIV‐)	2031
Growth‐positive samples processed	638 samples	164 specimens (120 HIV+, 44 HIV‐)	802
Isolates preserved for molecular work	164 isolates	164 isolates	328
16S rRNA PCR amplification	164 amplicons	164 amplicons	328
RFLP digestion (*Taq*I & *Cs6pI*)	164 profiles screened	164 profiles screened	328
Final sequenced representative Isolates	80 isolates	63 isolates (40 HIV+, 23 HIV‐)	143 isolates

### 2.4 Culture‐Based Isolation and Identification of Enteric Bacteria

Standard microbiological procedures were used to isolate *E. coli* as an indicator of fecal contamination and to detect selected enteric bacterial species, including *Salmonella* spp., *Shigella* spp., and *Vibrio* spp. Culture media included Chromocult Coliform Agar (CCA), Xylose Lysine Deoxycholate (XLD) agar, Tetrathionate Broth (TTB), Thiosulphate–Citrate–Bile Salts–Sucrose (TCBS) agar, and Alkaline Peptone Water (pH 8.5) (Merck, South Africa). All media were prepared according to the manufacturer's instructions and standard microbiological procedures (SABS 2001; APHA 2005).

For drinking water analysis, fecal coliforms were enumerated using membrane filtration techniques on mFC agar and CCA, followed by incubation at 44.5°C for 24 h. For the isolation of *Salmonella* and *Shigella* spp., 500 mL drinking water samples were filtered through sterile membrane filters (47 mm diameter; 0.45 μm pore size; Millipore). Following filtration, membranes were aseptically transferred into 50 mL of sterile Selenite broth (Merck, South Africa) and incubated aerobically at 36°C ± 1°C for 18–24 h. Subsequently, 1 mL of pre‐enriched suspension was inoculated into 9 mL TTB and incubated overnight at 36°C ± 1°C. Presumptive *Salmonella* and *Shigella* spp. were isolated on XLD agar following incubation at 36°C ± 1°C overnight. For *Vibrio* spp., water samples were pre‐enriched in 100 mL double‐strength alkaline peptone water (pH 8.5) and incubated at 36°C ± 1°C for 6–8 h prior to selective culture on TCBS agar. For stool specimens, approximately a pea‐sized aliquot (~0.5 g) was directly inoculated into the corresponding enrichment broths. All culture procedures were performed in triplicate.

For both drinking water and stool specimens, three to five presumptive colonies displaying distinct morphological characteristics (colony size, shape, and color) were selected per plate. Selected colonies were subcultured onto selective agar slants and incubated at 36°C ± 1°C for 24 h to obtain purified isolates.

### 2.5 Molecular Characterization and Species‐Specific PCR

For molecular analyses, nutrient broth and tryptic soy broth were inoculated with 328 bacterial isolates (164 drinking water, 164 stool) and incubated at 35°C ± 1°C. Genomic DNA was extracted using the ZR Fungal/Bacterial DNA MiniPrep kit (ZYMO Research, USA) and purified using the DNeasy DNA purification kit (QIAGEN, Germany) according to the manufacturers' instructions or the lysis protocol described by (Theron et al. 2000). DNA concentration and purity were assessed using a NanoDrop ND‐1000 spectrophotometer (Thermo Fisher Scientific). Universal bacterial primers 27F and 1507R were used for 16S rRNA gene amplification.

PCR reactions were performed in a total volume of 25 μL containing 12.5 μL DreamTaq MasterMix (2×) (Fermentas, St. Leon‐Rot, Germany), 0.5 μL Forward Primer (10 pmol), 0.5 μL Reverse Primer (10 pmol), 8.5 μL Nuclease‐free water, and 3.0 μL Template DNA. Amplification was performed in an MJ MINI thermal cycler (Bio‐Rad). Thermal cycling conditions consisted of an initial denaturation at 94°C for 10 min; 35 cycles of denaturation at 94°C for 30 s, annealing at 55°C for 30 s, and extension at 72°C for 30 s; followed by a final extension at 72°C for 7 min. Extraction blanks, positive control strains ( *E. coli* ATCC 25922, *S. Typhimurium* ATCC 14028, *Vibrio cholerae* ATCC 14035), and no‐template negative controls were included in each run.

Species‐specific PCR primers (Table 2) were used for molecular confirmation of selected isolates prior to sequencing. Note on pathotype terminology: Detection of the *uidA* gene confirms *E. coli* at the species level but does not establish enteropathogenic *E. coli* (EPEC) or specific diarrheagenic pathotypes without testing for specific virulence‐associated markers. Therefore, isolates are classified at the species level as *E. coli* .

**TABLE 2:** Primers used in species‐specific PCR analysis.

Organism	Target gene	Amplicon size (bp)	Primer sequence (5′‐3′)	References
*Salmonella typhimurium*	*EpaB*	314	**IpaB‐F**: GGACTTTTTAAAAGCGGCGG **IpaB‐R**: GCCTCTCCCAGAGCCGTCTGG	(Kong et al. 2002)
*Shigella dysenteriae*	*ipaH*	199	**ipaH‐U1**: CCTTTTCCGCGTTCCTTGA **ipaH‐L1**: CGGAATCCGGAGGTATTGC	(Thiem et al. 2004)
*Vibrio cholerae*	*EpsM*	248	**EpsM‐F**: GAATTATTGGCTCCTGTGCAGG **EpsM‐R**: ATCGCTTGGCGCATCACTGCC	(Kong et al. 2002)
*Escherichia coli*	*UidA*	147	**UAL‐754**: AAAACGGCAAGAAAAAGCAG **UAR‐900**: ACGCGTGGTTAACAGTCTTGC	(Tsai et al. 1993)

### 2.6 Restriction Analysis of PCR Amplicons (PCR‐RFLP)

To select representative isolates for sequencing and eliminate redundant clones, all 328 PCR amplicons were subjected to Restriction Fragment Length Polymorphism (RFLP) analysis. Briefly, 10 μL of each 16S rRNA PCR amplicon was digested using restriction enzymes *Taq*I and *Cs6pI* (Fermentas, Germany) at 65°C (*Taq*I) and 37°C (*Cs6pI*) for 2 h according to the manufacturer's instructions. Restriction fragments were separated on 1.5% (w/v) agarose gels stained with ethidium bromide and visualized under UV light. HyperLadder 1 kb (Bioline Products, South Africa) was included as a molecular size marker. Restriction profiles were analyzed manually. To reduce redundancy while ensuring profile representation, one representative isolate was selected for sequencing for every five isolates displaying identical restriction fragment patterns. Following RFLP profile selection, 143 representative isolates (80 drinking water, 63 stool) were selected for Sanger sequencing.

### 2.7 Phylogenetic Analysis

Edited nucleotide sequences were subjected to BLASTn analysis against the NCBI database for preliminary sequence confirmation. Sequences were aligned using Clustal W alongside reference sequences retrieved from GenBank. Phylogenetic analyses were conducted using Molecular Evolutionary Genetics Analysis (MEGA) version 6 (Tamura et al. 2013). Neighbor‐joining phylogenetic trees were constructed using the Kimura two‐parameter model with 1000 bootstrap replicates. For amplicons shorter than 500 bp, sequence alignment was used to evaluate genetic relatedness rather than formal tree construction.

### 2.8 Diversity Indices and Statistical Framework

Sequences were clustered into Operational Taxonomic Units (OTUs) (Sneath and Sokal 1973) using MOTHUR (Schloss et al. 2009) at a ≥ 97% sequence identity threshold based on 16S rRNA gene sequence similarity. Alpha diversity was evaluated using Shannon, Simpson, Chao, and ACE estimators to evaluate sequence‐level variation among the selected representative isolates across sample matrices. Because diversity indices were derived from selected representative isolates rather than whole‐community metagenomic libraries, these indices are presented as descriptive measures of sequence‐level variation rather than inferential community ecology dynamics.

## 3 Results

### 3.1 Prevalence of Enteric Organisms in Drinking Water Samples and in Stool Specimens

Figure 2 indicates data derived from growth‐positive container‐stored drinking water samples (*n* = 638) collected across Boboyi, Bomela, and Gamalakhe. Targeted bacterial taxa include species‐level *E. coli* (uidA gene target) and presumptive *Salmonella* spp., *Shigella* spp., and *Vibrio* spp. Pathogen prevalence differed among the three sampling locations. Drinking water samples from Boboyi showed detection frequencies above 80% for *Salmonella* spp., *Shigella* spp., *Vibrio* spp., and *E. coli* , whereas samples from Bomela showed detection frequencies below 60% for all target organisms. Gamalakhe recorded intermediate detection frequencies of 60%–75% for *Salmonella* spp., *Shigella* spp., and *Vibrio* spp., ranged between 60% and 75%, whereas *E. coli* detection frequency was below 60%, demonstrating noticeable spatial variation in household container‐stored drinking water quality across the study communities.

**FIGURE 2:**
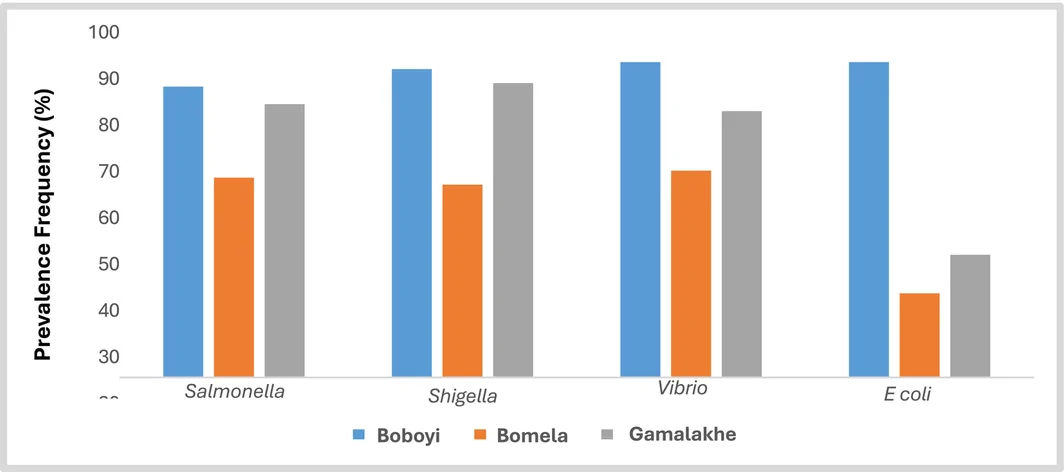
Prevalence frequency (%) of presumptive enteric bacteria in container‐stored drinking water across study communities. [Data derived from growth‐positive container‐stored drinking water samples (*n* = 638) collected across Boboyi, Bomela, and Gamalakhe. Targeted bacterial taxa include species‐level *Escherichia coli* (uidA gene target) and presumptive Salmonella spp., Shigella spp., and Vibrio spp.]

Figure 3 presents the prevalence of enteric bacterial pathogens detected in diarrheal stool specimens according to HIV status. Sample denominators include household container‐stored drinking water (*n* = 638 growth‐positive samples) and diarrheal stool specimens (*n* = 164 participants: *n* = 120 HIV‐infected individuals, *n* = 44 HIV‐negative controls). Target organisms include species‐confirmed *E. coli* (*uidA* gene target) and presumptive *Salmonella* spp., *Shigella* spp., and *Vibrio* spp. Marked differences in pathogen prevalence were observed between HIV‐positive and HIV‐negative individuals. *E. coli* was detected in 88% of stool specimens collected from HIV‐positive individuals compared with 34% among HIV‐negative controls. Stool specimens from HIV‐positive individuals showed prevalence values of 33%, 27%, and 12% for *Salmonella* spp., *Shigella* spp., and *Vibrio* spp., respectively. Among HIV‐negative individuals, *Shigella* spp. and *Vibrio* spp. were not detected, whereas *Salmonella* spp. was detected at a prevalence of 2%.

**FIGURE 3:**
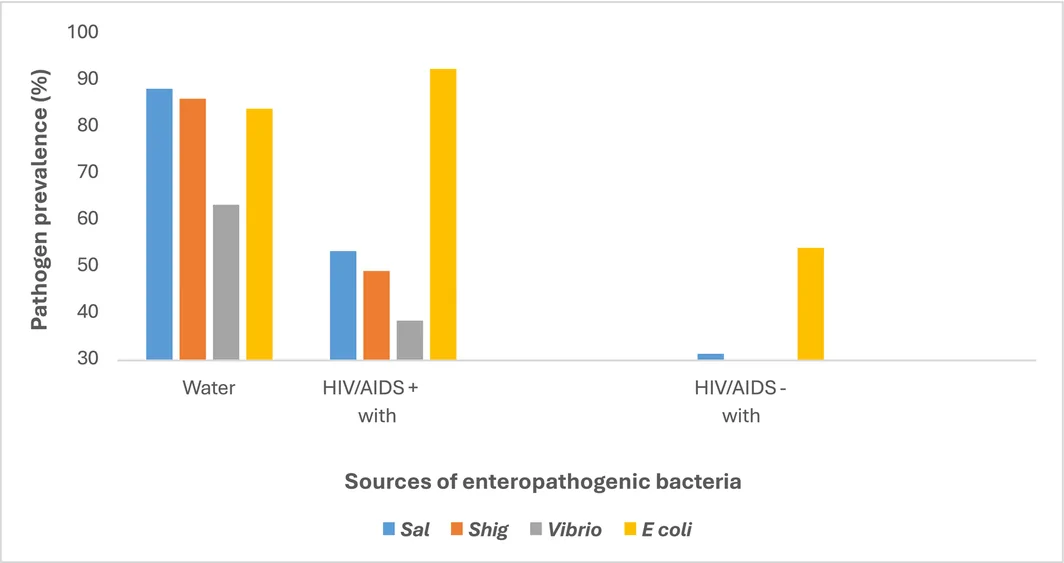
Prevalence comparison of enteric bacteria between household container‐stored drinking water and diarrheal stool specimens. [Sample denominators: household container‐stored drinking water (*n* = 638 growth‐positive samples) and diarrheal stool specimens (*n* = 164 participants: *n* = 120 HIV‐infected individuals, *n* = 44 HIV‐negative controls). Target organisms include species‐confirmed *Escherichia coli* (uidA gene target) and presumptive Salmonella spp., Shigella spp., and Vibrio spp.]

### 3.2 Molecular Identification of Bacteria Detected in Drinking Water Samples and Stool Specimens

Genomic DNA extracted from presumptive bacterial isolates obtained from container‐stored drinking water samples was subjected to PCR amplification targeting the 16S rRNA gene. Figure 4 illustrates a representative agarose gel electrophoresis (1.0% w/v) showing 16S rRNA gene PCR amplicons (~1500 bp) obtained from selected drinking water isolates. Distinct, sharp band profiles of the expected size (~1500 bp) were observed across representative lanes (Lanes 1–10 and Lanes 11–19), confirming successful amplification against the FastRuler Low Range DNA Molecular Weight Ladder (1 kb; Lane M) prior to downstream RFLP fingerprinting and sequencing. Figure 5 displays a representative agarose gel electrophoresis (1.0% w/v) showing 16S rRNA gene PCR amplicons (~1500 bp) obtained from selected clinical stool isolates. Clear, specific bands of the expected size (~1500 bp) were consistently observed across representative lanes (Lanes 2–19) from HIV‐infected patients when evaluated against the DNA molecular weight ladder (1 kb; Lane 1), confirming successful amplicon recovery before downstream RFLP fingerprinting and Sanger sequencing.

**FIGURE 4:**
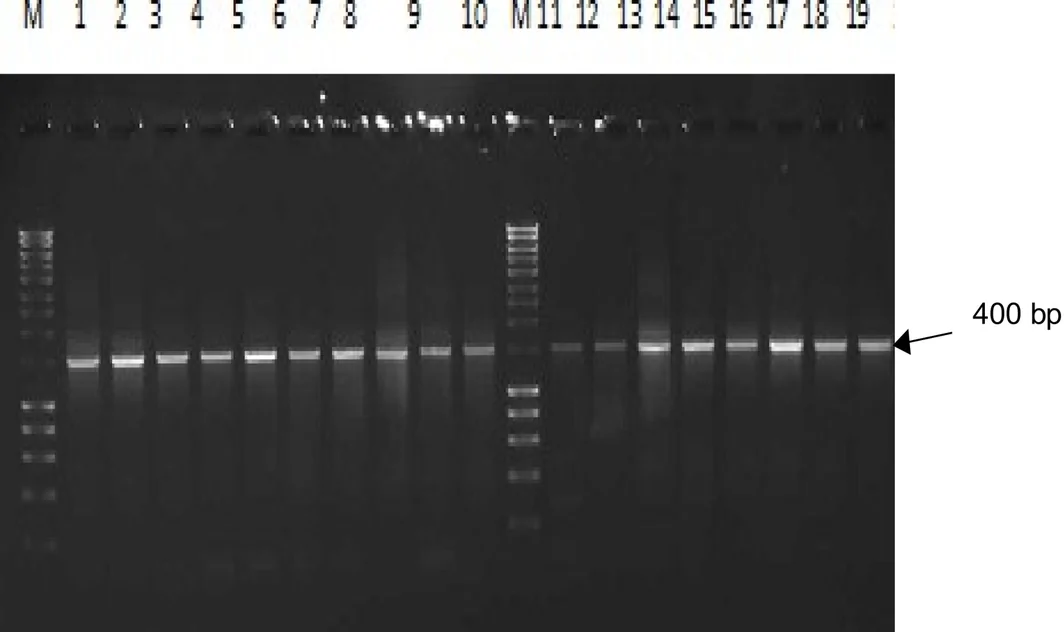
Representative agarose gel electrophoresis (1.0% w/v) of genomic DNA and 16S rRNA gene PCR amplicons (~1500 bp) from selected drinking water bacterial isolates. [Lanes 1–10 and Lanes 11–19 represent amplified 16S rRNA gene products from representative container‐stored drinking water isolates. Lane M: FastRuler Low Range DNA Molecular Weight Ladder (1 kb)].

**FIGURE 5:**
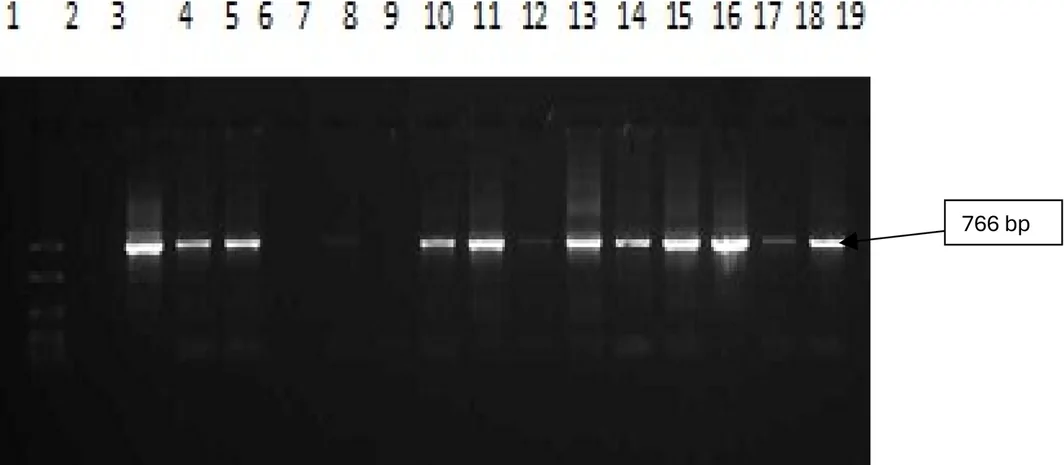
Representative agarose gel electrophoresis (1.0% w/v) of genomic DNA and 16S rRNA gene PCR amplicons (~1500 bp) from selected clinical stool specimens. [Lanes 2–19 represent 16S rRNA gene PCR amplicons derived from diarrheal stool specimens of HIV‐infected patients. Lane 1: DNA molecular weight ladder (1 kb)].

To eliminate redundant sequence analysis of clonal replicates, 16S rRNA gene PCR amplicons (~1500 bp) obtained from growth‐positive drinking water isolates were subjected to restriction fragment length polymorphism (RFLP) digestion using restriction endonucleases TaqI (65°C, 2 h) and Csp6I/Cs6pI (37°C, 2 h). Figure 6 illustrates representative RFLP fingerprint profiles separated on a 1.5% (w/v) agarose gel alongside a 1 kb DNA molecular weight ladder (Lane 1). Digestion profiles across representative lanes (Lanes 2–19) were evaluated to group identical restriction patterns, leading to the selection and retention of 80 representative water‐derived isolates (*n* = 80) for downstream Sanger sequencing and phylogenetic characterization. Similarly, 16S rRNA gene amplicons derived from clinical stool specimens were analyzed by RFLP to assess pattern diversity among clinical bacterial isolates. Figure 7 shows representative RFLP digestion profiles obtained following dual restriction endonuclease cleavage with TaqI (65°C, 2 h) and Csp6I/Cs6pI (37°C, 2 h) on a 1.5% (w/v) agarose gel. Banding patterns across representative lanes (Lanes 2–19) compared against the 1 kb DNA ladder (Lane 1) allowed for the identification of unique restriction groups, resulting in 63 representative stool isolates (*n* = 63: 40 from HIV‐infected individuals and 23 from HIV‐negative controls) being selected for Sanger sequencing.

**FIGURE 6:**
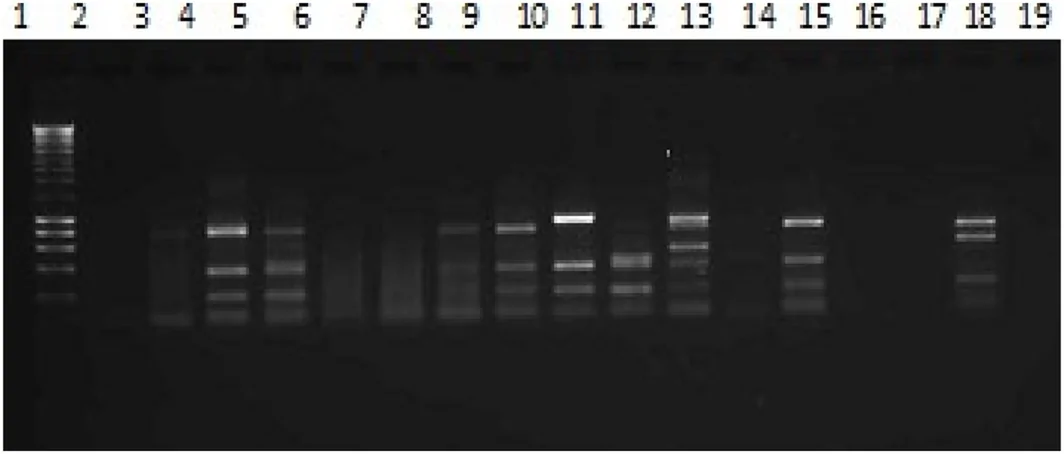
Restriction fragment length polymorphism (RFLP) fingerprint profiles of 16S rRNA gene amplicons from drinking water bacterial isolates. [These PCR amplicons were digested using restriction endonucleases TaqI (65°C, 2 h) and Csp6I/Cs6pI (37°C, 2 h) and separated on a 1.5% (w/v) agarose gel. Lane 1: 1 kb DNA molecular weight ladder; Lanes 2–19: RFLP digestion profiles used to group matching patterns and select representative isolates for Sanger sequencing (*n* = 80 water isolates retained)].

**FIGURE 7:**
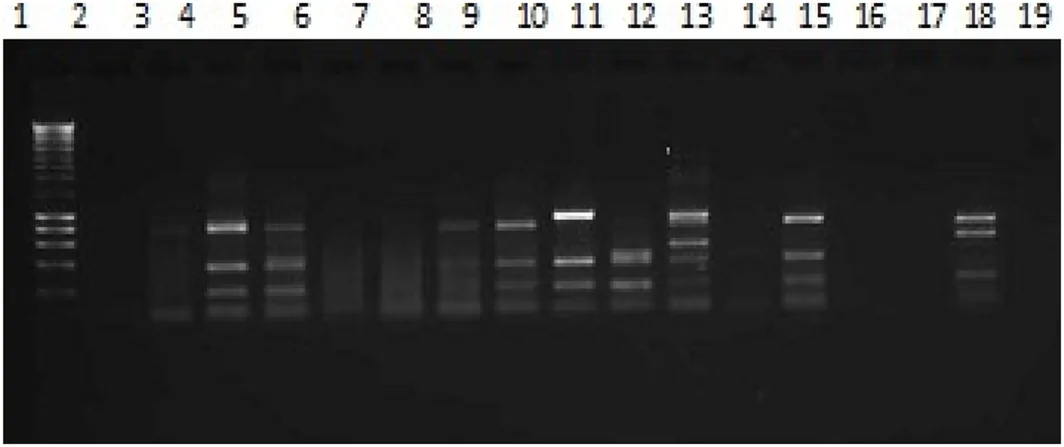
Restriction fragment length polymorphism (RFLP) fingerprint profiles of 16S rRNA gene amplicons from clinical stool‐derived bacterial isolates. [These PCR amplicons were digested using restriction endonucleases TaqI (65°C, 2 h) and Csp6I/Cs6pI (37°C, 2 h) and separated on a 1.5% (w/v) agarose gel. Lane 1: 1 kb DNA molecular weight ladder; Lanes 2–19: RFLP fingerprint patterns of clinical stool isolates (*n* = 63 stool isolates retained)].

Of the 328 isolates subjected to molecular analysis, 143 representative isolates were selected for sequencing following restriction profile analysis, comprising 80 isolates recovered from drinking water samples and 63 isolates recovered from stool specimens. Sequence data generated from representative isolates were compared against reference sequences available in the GenBank database using BLASTn analysis. The sequence comparisons enabled molecular identification and assignment of the selected isolates to bacterial taxa corresponding to *E. coli* , *Salmonella enterica* , *Shigella dysenteriae* , and *V. cholerae* .

### 3.3 Genetic Relatedness of Bacterial Isolates

Genetic relatedness analyses were performed to evaluate relationships among bacterial isolates recovered from drinking water and stool specimens. Phylogenetic analysis was conducted only for *E. coli* isolates, as sufficient sequence lengths were available for tree construction. For *Vibrio* and *Salmonella*, sequence lengths shorter than 500 bp limited phylogenetic reconstruction; therefore, a multiple sequence alignment generated using Clustal W was used to assess genetic relatedness. In addition, limited sequence representation for *Shigella* (*ipaH*), *Salmonella* (*ipaB*), and *Vibrio* (*epsM*) restricted comparative phylogenetic analysis, and BLASTn sequence comparisons were therefore used for taxonomic comparison. Phylogenetic relationships were inferred using the neighbor‐joining method (Saitou and Nei 1987) and the Kimura two‐parameter model (Kimura 1980) with bootstrap support based on 1000 replicates (Felsenstein 1989).

To evaluate the genetic evolutionary relatedness between environmental drinking water isolates and clinical stool isolates, a Neighbor‐Joining phylogenetic tree was constructed based on near full‐length 16S rRNA gene sequences (> 500 bp) using MEGA 6.0. Figure 8 illustrates the phylogenetic tree constructed using the Kimura 2‐parameter evolutionary model with 1000 bootstrap replicates. High sequence similarity and tight phylogenetic clustering were observed between environmental container‐stored drinking water isolates (e.g., SK1‐Bys and SK1‐Bms) and clinical diarrheal stool isolates (e.g., 44 SK1, 64 SK1, 161 SK1, and 162 SK1) from Ugu District, forming supported clades alongside GenBank reference strains. Branch node values indicate percentage bootstrap support across 1000 replications, and the scale bar represents nucleotide substitutions per site.

**FIGURE 8:**
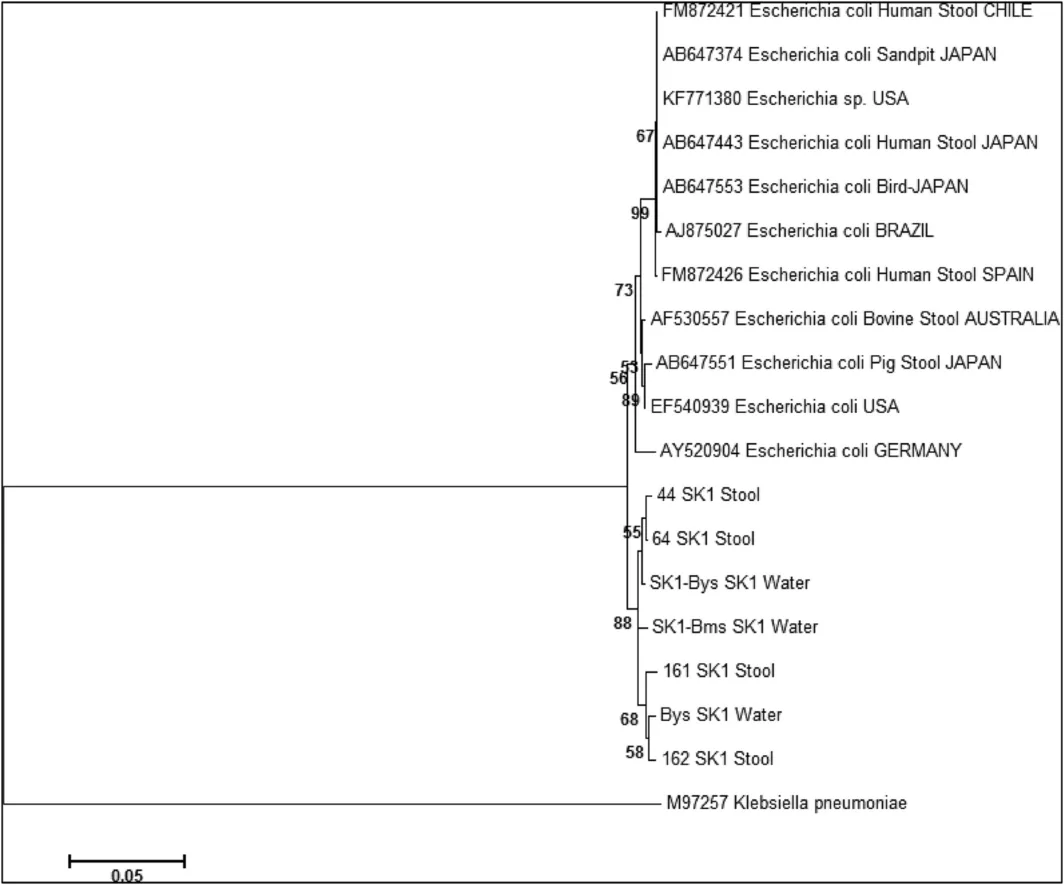
Neighbor‐Joining phylogenetic tree based on 16S rRNA gene sequences (> 500 bp) showing genetic relatedness between selected drinking water and clinical stool‐derived *Escherichia coli* isolates. [Reconstructed in MEGA 6.0 using the Kimura 2‐parameter evolutionary model. Percentage bootstrap support values (1000 replications) are shown at node branches. Environmental water isolates (e.g., SK1‐Bys and SK1‐Bms) and clinical stool isolates (e.g., 44 SK1, 64 SK1, 161 SK1, and 162 SK1) from Ugu District cluster closely with GenBank reference strains. Scale bar indicates nucleotide substitutions per site].

Figure 9 presents the Clustal W multiple sequence alignment of Vibrio spp. target gene fragments (epsM and CTX) recovered from household container‐stored drinking water and clinical diarrheal stool specimens alongside *V. cholerae* reference sequences retrieved from GenBank. Because sequence lengths were shorter than 500 bp, formal phylogenetic tree construction was not performed, and genetic comparison was evaluated using multiple sequence alignment. The alignment demonstrated substantial sequence conservation among clinical stool isolates, environmental water isolates, and GenBank reference sequences. Stool‐derived Vibrio sequences aligned closely with reference strains, whereas water‐derived isolates exhibited limited sequence variation characterized by a small number of single‐nucleotide polymorphisms (SNPs) within otherwise conserved regions. Dots indicate nucleotide positions identical to the reference sequence.

**FIGURE 9:**
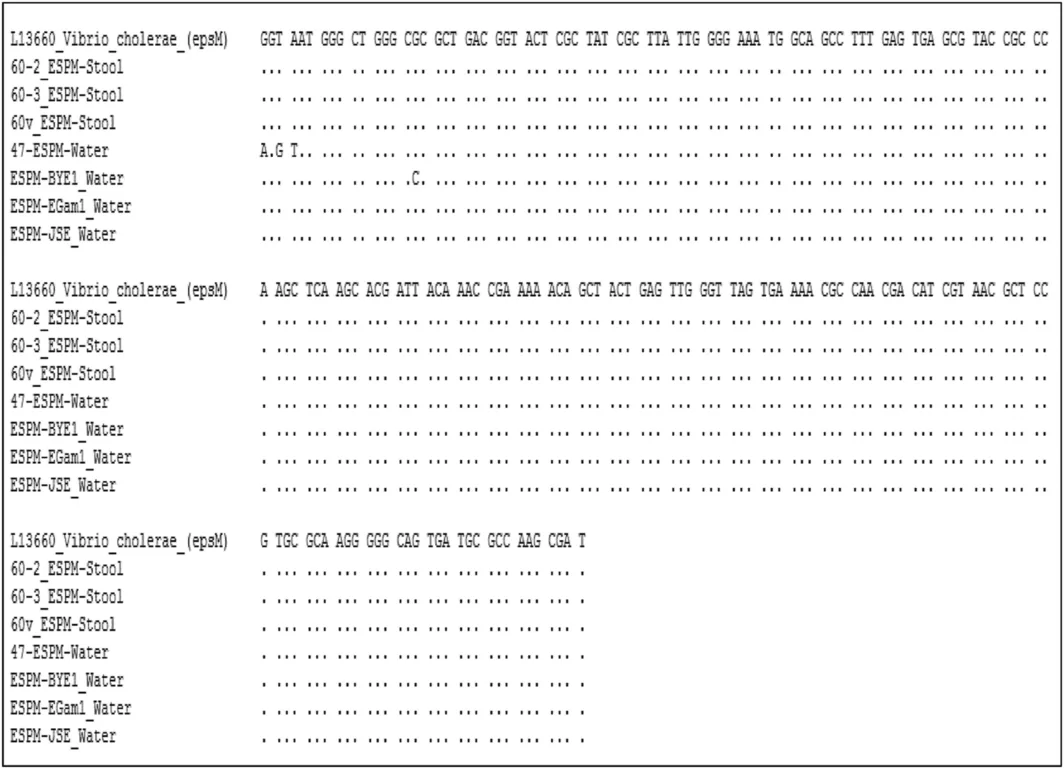
Clustal W multiple sequence alignment of Vibrio spp. target gene fragments (< 500 bp) comparing environmental water isolates, clinical stool isolates, and GenBank reference strains. [Multiple sequence alignment comparing short sequence amplicons (epsM and CTX gene targets) of Vibrio isolates recovered from household container‐stored drinking water and clinical diarrheal stool specimens against *Vibrio cholerae* reference sequences from GenBank. Dots indicate conserved nucleotide positions across environmental, clinical, and reference sequences].

Figure 10 displays the Clustal W multiple sequence alignment of Salmonella spp. target gene fragments (epaB and invA) recovered from household container‐stored drinking water and clinical diarrheal stool specimens alongside *Salmonella enterica* reference sequences retrieved from GenBank. Similar to the analysis for Vibrio, sequence lengths below 500 bp precluded formal phylogenetic tree construction, so genetic comparison was evaluated using multiple sequence alignment. The alignment demonstrated high nucleotide sequence conservation across clinical stool isolates, environmental water isolates, and GenBank reference strains, with minimal sequence variation observed across the target region. Dots indicate nucleotide positions identical to the reference sequence.

**FIGURE 10:**
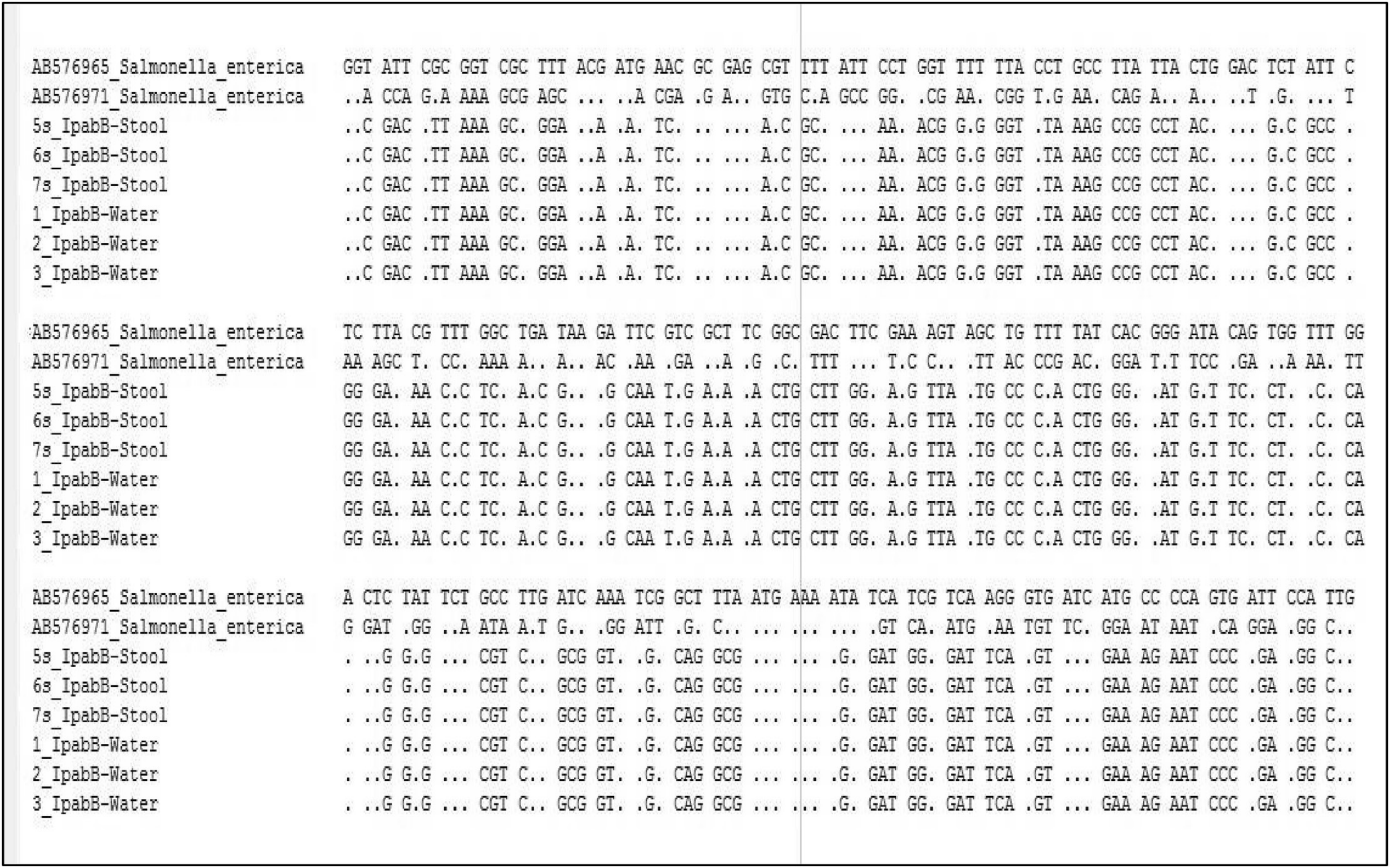
Clustal W multiple sequence alignment of Salmonella spp. target gene fragments (< 500 bp) comparing environmental water isolates, clinical stool isolates, and GenBank reference strains. [Multiple sequence alignment comparing short sequence amplicons (epaB and invA gene targets) of Salmonella isolates recovered from household container‐stored drinking water and clinical diarrheal stool specimens against *Salmonella enterica* reference sequences from GenBank. Dots indicate conserved nucleotide positions across environmental, clinical, and reference sequences].

### 3.4 Alpha Diversity Indices

Tables 3 and 4 present the alpha diversity estimates calculated from sequence data obtained from bacterial isolates recovered from drinking water and stool specimens. Diversity assessment was performed using the Shannon and Simpson diversity indices together with richness estimators including Chao and ACE to evaluate sequence‐level variation among the selected isolates. Variation in sequence diversity was observed between isolates recovered from stool specimens and those recovered from drinking water. For *Salmonella*, stool‐derived isolates showed higher Shannon and ACE values than water‐derived isolates. Similarly, *Shigella* isolates recovered from stool specimens showed higher diversity estimates than their water‐derived counterparts. In contrast, *Vibrio* isolates showed comparatively lower diversity values across both sample sources.

**TABLE 3:** Diversity indices of genes across target bacterial gene sequences from combined isolate collections.

Target organism	Gene	Source	No. of sequences	No. of unique OTUs	Shannon index	Simpson index	Chao	ACE	Jackknife
*E. coli*	*SK1*	Combined	14	9	2.069	0.077	16.5	17.97	20.71
*Salmonella*	*invA*	Combined	19	9	2.041	0.099	11.0	12.08	13.00
*Salmonella*	*ipaB*	Combined	44	19	2.392	0.136	110.0	110.19	168.76
*Salmonella*	*mdh31*	Combined	8	5	1.386	0.214	11.0	34.82	14.50
*Shigella*	*mdh50*	Combined	16	13	2.480	0.033	40.5	55.21	49.99
*Vibrio*	*CTX*	Combined	11	11	1.999	0.000	66.0	0.00	22.00
*Vibrio*	*epsM*	Combined	18	12	2.370	0.052	21.33	23.46	21.11

*Note:* OTU = Operational Taxonomic Unit (defined at ≥ 97% sequence similarity threshold); ACE = Abundance‐based Coverage Estimator.

**TABLE 4:** Diversity indices of genes comparing stool‐derived and water‐derived bacterial isolates individually.

Target organism	Gene	Source	No. of sequences	No. of unique OTUs	Shannon index	Simpson index	Chao	ACE	Jackknife
*E. coli*	*SK1*	Stool	11	6	1.673	0.127	7.50	8.46	9.00
		Water	35	6	1.029	0.491	7.50	16.95	9.00
*Salmonella*	*invA*	Stool	4	2	0.693	0.333	2.00	2.00	2.00
		Water	15	8	1.899	0.114	18.00	14.79	28.03
*Salmonella*	*ipaB*	Stool	21	13	2.359	0.071	58.00	59.69	45.44
		Water	23	7	1.011	0.304	10.00	22.99	11.00

*Note:* OTU = Operational Taxonomic Unit (defined at ≥ 97% sequence similarity threshold); ACE = Abundance‐based Coverage Estimator.

## 4 Discussion

The recovery of *E. coli* , *Salmonella* spp., *Shigella* spp., and *Vibrio* spp. from both drinking water and stool specimens indicates the continued presence of enteric bacterial contamination within the study setting. The occurrence of these organisms in environmental and clinical samples remains important from a public health perspective, particularly among HIV‐infected individuals and other populations with increased susceptibility to infection. Similar observations have been reported in studies linking poor household water quality and unsafe water storage practices with increased exposure to enteric pathogens and gastrointestinal disease.

It is important to clarify the pathotype terminology used in this study. Detection of the *uidA* gene confirms *E. coli* at the species level but does not establish enteropathogenic *E. coli* (EPEC) or specific diarrheagenic pathotypes without testing for specific virulence‐associated markers. Therefore, isolates in this study are classified at the species level as *E. coli* or broadly as enteric bacteria, avoiding the overextension of species‐level identification to confirmed enteropathogenicity.

Molecular analyses provided additional resolution beyond culture‐based detection by enabling characterization at the sequence level (Theron and Cloete 2002; Adzitey and Huda 2010; Yashiro et al. 2011; Stefani et al. 2015; Donelli et al. 2013). Sequence identification showed that isolates recovered from drinking water and stool specimens corresponded to recognized enteric species and displayed varying levels of genetic relatedness (Islam et al. 1998; Lampel et al. 2000; Kulkarni et al. 2002; Tsen and Jian 1998; Waage et al. 1999; Rood et al. 2011; Salama et al. 2012; Njage and Buys 2015; Nandi et al. 2000; Goel et al. 2007). Similar studies have reported improved discrimination and bacterial characterization when molecular approaches are incorporated into investigations of enteric pathogens.

Phylogenetic analysis of *E. coli* demonstrated close clustering between selected drinking water and stool‐derived isolates, suggesting genetic relatedness between environmental and clinical strains identified within the study area (Tsen and Jian 1998; Waage et al. 1999; Rood et al. 2011; Salama et al. 2012; Njage and Buys 2015). Crucially, while sequence similarity and phylogenetic clustering demonstrate genetic relatedness, they do not prove direct water‐to‐human transmission, a common contamination source, or definitive epidemiological linkage. Short sequence fragments derived from conserved targets (such as the 16S rRNA gene) provide species confirmation and broad genetic classification but lack the discriminatory resolution required to trace direct dissemination pathways. Container‐stored household drinking water should therefore be interpreted as a potential environmental exposure source rather than a proven direct transmission vector. To definitively demonstrate common‐source transmission or direct water‐to‐human dissemination, high‐resolution strain‐level typing approaches such as Multi‐Locus Sequence Typing (MLST) or Whole‐Genome Sequencing (WGS) are necessary (Davies et al. 2020; Murei and Momba 2025).

In contrast, phylogenetic reconstruction for *Salmonella* spp., *Shigella* spp., and *Vibrio* spp. could not be fully achieved because sequence lengths were shorter than 500 bp and did not support robust tree construction. Sequence alignment using Clustal W was therefore used to assess relatedness among isolates. Conserved sequence regions were observed between study isolates and reference sequences retrieved from GenBank, indicating similarity to previously reported strains, although sequence length reduced confidence in phylogenetic interpretation.

Diversity indices were applied to evaluate sequence variation among isolates recovered from different sources (Sneath and Sokal 1973; Schloss et al. 2009; Felsenstein 1989). Although these indices are commonly used in microbial community studies, they were applied here as descriptive measures of sequence‐level variation among selected, sequence‐confirmed representative isolates rather than inferential community‐wide ecological dynamics. Stool‐derived *Salmonella* spp. and *Shigella* spp. isolates showed greater diversity than corresponding drinking water isolates, whereas *Vibrio* spp. isolates demonstrated lower diversity across both sources.

## 5 Study Limitations

The interpretation of the genetic and environmental findings must consider several clinical, environmental, and analytical limitations:

1. Unmeasured Clinical Confounders: Because patient clinical records were anonymized to protect participant confidentiality, key clinical variables including CD4 + T‐cell counts, viral load, antiretroviral therapy (ART) compliance, concurrent antimicrobial treatment, duration and severity of diarrhea, patient age, and sex were not recorded (UNAIDS 2012; Guerrant et al. 2002; Wilson et al. 2010; Obi et al. 2007). These clinical parameters significantly influence host susceptibility, stool pathogen shedding, and disease severity in HIV‐infected populations.
2. Behavioral and Environmental Confounders: Detailed household‐level data regarding sanitation facility design, latrine proximity to water storage, personal hygiene practices, food preparation safety, and alternative drinking water sources were not recorded for all participants.
3. Molecular Typing Resolution: Short sequence lengths restricted phylogenetic analysis for selected bacterial groups and required reliance on sequence alignment rather than complete phylogenetic reconstruction. Molecular evaluations relied on 16S rRNA and species‐specific single‐gene PCR amplicons (Lane 1991; Heyndrickx et al. 1991; Kong et al. 2002; Thiem et al. 2004; Tsai et al. 1993). While these targets confirm species identity and broad similarity, strain‐level typing (MLST or WGS) is required to establish true clonal identity and epidemiological transmission chains.

Despite these limitations, the genetic relatedness observed between selected environmental and clinical isolates supports the continued use of molecular tools in studies investigating water‐associated enteric infections. Although the findings do not establish direct transmission pathways, they suggest that household container‐stored drinking water should remain an important consideration when evaluating exposure to enteric bacterial pathogens in vulnerable populations.

## 6 Conclusion

This study demonstrated the occurrence of enteric bacteria in household container‐stored drinking water and diarrheal stool specimens of HIV‐infected individuals in KwaZulu‐Natal and identified varying levels of genetic relatedness among selected isolates recovered from environmental and clinical sources. Molecular characterization and sequence‐based analyses provided additional discriminatory capacity beyond conventional culture‐based methods and contributed to improved understanding of relationships among bacterial isolates recovered from the two sample types. Although direct transmission pathways could not be confirmed, the observed genetic relatedness between selected environmental and clinical isolates indicates that household drinking water should remain an important consideration when investigating enteric bacterial exposure. These findings support continued efforts to improve household water quality and strengthen the application of molecular approaches in studies investigating water‐associated enteric infections in vulnerable populations.

## Author Contributions

**C. M. N. Khabo‐Mmekoa:** investigation, methodology, validation, visualization, writing – original draft, writing – review and editing. **K. Jitendra:** methodology, writing – review and editing, formal analysis. **M. N. B. Momba:** conceptualization, funding acquisition, validation, visualization, writing – review and editing, supervision.

## Funding

The South African Research Chairs Initiative (SARChI Chair in Water Quality and Health Management) funded this study (UID87310).

## Conflicts of Interest

The authors declare no conflicts of interest.

## Data Availability

The datasets generated and/or analyzed during the current study are available from the corresponding author upon reasonable request.
